# Immune-related gene signature associates with immune landscape and predicts prognosis accurately in patients with skin cutaneous melanoma

**DOI:** 10.3389/fgene.2022.1095867

**Published:** 2023-01-04

**Authors:** Xin Shen, Lifeng Shang, Junwei Han, Yi Zhang, Wenkai Niu, Haiwang Liu, Hai Shi

**Affiliations:** Department of Gastrointestinal Surgery, Xi’an Daxing Hosptial, Xi’an, China

**Keywords:** skin cutaneous melanoma, immune pathway, prognosis, bioinformatics, tumor environment

## Abstract

Skin cutaneous melanoma (SKCM) is the skin cancer that causes the highest number of deaths worldwide. There is growing evidence that the tumour immune microenvironment is associated with cancer prognosis, however, there is little research on the role of immune status in melanoma prognosis. In this study, data on patients with Skin cutaneous melanoma were downloaded from the GEO, TCGA, and GTEx databases. Genes associated with the immune pathway were screened from published papers and lncRNAs associated with them were identified. We performed immune microenvironment and functional enrichment analyses. The analysis was followed by applying univariate/multivariate Cox regression algorithms to finally identify three lncRNAs associated with the immune pathway for the construction of prognostic prediction models (CXCL10, RXRG, and SCG2). This stepwise downscaling method, which finally screens out prognostic factors and key genes and then uses them to build a risk model, has excellent predictive power. According to analyses of the model’s reliability, it was able to differentiate the prognostic value and continued existence of Skin cutaneous melanoma patient populations more effectively. This study is an analysis of the immune pathway that leads lncRNAs in Skin cutaneous melanoma in an effort to open up new treatment avenues for Skin cutaneous melanoma.

## Introduction

Melanoma can occur in the skin, mucosa and ocular choroid, with SKCM accounting for 91% of cases (Dunn et al., 2016; Yang et al., 2016). The disease is closely associated with Sun exposure; it is partly associated with trauma, immune deficiency and viral infections (Carr et al., 2019). It has a low incidence, is prone to early metastasis and has a very high mortality rate. Analysis of global cancer statistics has shown a significant increase in the incidence and mortality of SKCM in recent years (Sung et al., 2021), which accounts for only 5% of all cutaneous malignancies but is willing to take responsibility for 75 percentage points of skin tumor-related deaths (Siegel et al., 2021). The incidence of SKCM is estimated to be about 1/10th that of Non-Melanoma Skin Cancer (NMSC), but its death toll is 8 times higher than that of NMSC (Liu-Smith et al., 2017), with approximately 55,000 deaths from SKCM worldwide each year (Lodde et al., 2020). Metastases, which are difficult to treat and have a poor prognosis, occur in approximately 20% of patients diagnosed with early SKCM. The 5-year survival rate for patients with metastatic SKCM is only 15%–20% (Keilholz et al., 2020; Patel et al., 2020). In order to diagnose and treat early, the search for novel SKCM biomarkers and the establishment of risk modelling are necessary to improve early diagnosis, predict prognosis and guide clinical treatment. There are more treatment options for SKCM, of which surgery is the primary treatment, but complete surgical resection is required as residual cancer cells are the main cause of tumour recurrence and metastasis (Testori et al., 2016; Wen et al., 2020). In addition the tumour microenvironment (TME) is thought to have a huge impact on the behaviour and characteristics of cancer. The TME consists of non-cellular components such as the extracellular matrix and signalling molecule types and non-tumour cell components such as epithelial cells, smooth muscle cells and immune cells in the tumour ecological niche, and Zhang’s study found that crosstalk between tumour and non-tumour cells played an active role in regulating cancer development and treatment response (Zhang et al., 2020). In recent years, with the development of targeted therapies and immunotherapy, some progress has been made in the treatment of malignant SKCM (Emens et al., 2017; Zaremba et al., 2020; Korn et al., 2021), such as immune checkpoint inhibitors (Herbst et al., 2019), peripatetic cell therapy and tumour vaccines (Qi et al., 2015), which have achieved better efficacy in clinical trials, bringing new hope for the treatment of SKCM patients, in which tumour-infiltrating immune cells are expected to be a new prognostic marker (Goeppert et al., 2013; Kirilovsky et al., 2016), While studies in zebrafish by Elena Gómez-Abenza et al. showed that SPINT1 can try to control changes in the cellular immune microenvironment (TME) of SKCM (Gómez-Abenza et al., 2019), Min Yan et al. (2021). Actually pointed to a unique role for T cells in SKCM based on single-cell sequencing results. As a result, the current study will look into changes in the immune response of SKCM patients.

Previous studies on the composition of immune cells have mostly been done by flow cytometry or immunohistochemistry. Flow cytometry requires the breakdown of tissue into single cell suspensions, which can lead to the loss of important samples, and has many steps and high operating conditions, all of which can affect the results of the analysis; immunohistochemistry is susceptible to factors such as antibody incubation time and antibody concentration.

The maturation of gene microarray and sequencing technologies has generated biological big data, which has enabled a further leap in the understanding of diseases from traditional pathology to the genetic level, facilitating the birth and development of precision medicine. In this study, based on the microarray data of SKCM and normal skin transcriptional gene expression profiles from TCGA, GTEx, and GEO databases, bioinformatics was applied to explore the differential gene expression characteristics in SKCM and provide ideas for clinical precision treatment.

In this study, we aimed to develop a transcriptomics-based approach to reveal the state of immune cell activation and predict survival outcomes of SKCM patients. We collected three sets of transcriptional profiling data and corresponding clinical information from TCGA, GTEx, and GEO databases, explored the level of immune cell activation in melanoma by obtaining differential genes at the expression level, constructed a prognostic model for SKCM, and identified multiple differential genes associated with immune activation as potential biomarkers. In addition, we performed a comprehensive analysis of the risk model, including functional enrichment, immune activation, and immune infiltration. In short, our findings reveal a critical role for immune activation in melanoma, and we propose a convenient approach to help diagnose and predict survival outcomes in melanoma patients.

## Materials and methods

### Data collection

Gene expression data for SKCM were downloaded from the TCGA database (http://portal.gdc.cancer.gov/) for a total of 472 cases and 807 normal skin tissue samples. Clinical data of SKCM samples including age, sex, survival time, survival status, tumour stage and TNM stage were also downloaded. Two microarray datasets exploring the gene expression profile of human SKCM, GSE15605 and GSE46517, were obtained by searching the GEO (https://www.ncbi.nlm.nih.gov/geo/) database for “Skin Cutaneous Melanoma.” The microarray data for GSE15605 is based on the GPL570 platform and includes skin biopsies from 58 SKCM patients and 16 normal controls, while the microarray data for GSE46517 is based on the GPL96 platform and includes skin biopsies from 104 SKCM patients and eight normal controls. The immune pathway gene set was retrieved from previous relevant studies (Galon et al., 2013; Nirmal et al., 2018). The data were preprocessed as follows: the downloaded dataset was firstly, the probes corresponded to the genes, the null probes were removed and multiple probes corresponded to the same gene we then selected the median of them as the expression level of the gene.

### Extraction of relevant differentially expressed genes (DEGs)

Integrate the TCGA-SKCM and GTEX SKIN datasets, remove batch effects from both sets of data using the COMBAT function, and then do differential analysis on tumour samples from TCGA-SKCM and normal samples from GTEX SKIN and datasets GSE15605 and GSE46517 using the R package edgeR; online analysis tool geo2r to screen for differential genes using the geo database (https://www.nvbi.nlm.nih.gov/geo/Geo2r/). The limma package in R (Smyth et al., 2005) was used to screen DEGs. A threshold of FDR <0.1 was set, and to visualize gene expression, all genes in the two datasets were plotted separately as volcano plots. For the three datasets, Venn Diagrams of two sets of differential genes were drawn according to the screening criteria using the software Draw Venn Diagram (http://bioinformatics.psb.ugent.be/webtools/Venn/) to screen from the three datasets for common DEGs and to draw in R visualised heat map.

### Consensus clustering of subtypes based on DEGs in SKCM

Up-regulated gene molecules were analysed using the Consensus Cluster Plus package to identify SKCM subtypes (Gravendeel et al., 2009). NMF hierarchical clustering has been conducted on an adapted uniform dataset with k values ranging from 2 to 9, and the k value with the best cluster stability was chosen based on the clustering effect. Consensus matrices (CM) and CDF curves of consensus scores were used to determine the optimal number of clusters. The t-distributed stochastic neighbor embedding (t-SNE) method was used to validate the isoform assignment of mRNA expression information corresponding to immune-related genes (Zhou et al., 2018). Box plots and heat maps depicting the expression levels of these 16 genes in the two subtypes were used to investigate the differences in survival data between these two groups with survival curves.

### Estimation of the immune microenvironment in two groups of melanomas

Gene expression data from 472 melanoma tissues were extracted from mRNA expression data, data corrected using the R software limma package, and the abundance of infiltrating immune cells in each sample was estimated by single sample gene set enrichment analysis (R package GSVA) (Zhang et al., 2021; Xu et al., 2022a) and the MCPcounter algorithm. M1 macrophages, M2 macrophages, M0 macrophages, follicular helper T lymphocytes, unactivated CD4^+^ memory T lymphocytes, activated CD4^+^ memory T lymphocytes, γδ T lymphocytes, CD8^+^ T lymphocytes, regulatory T lymphocytes, naive CD4^+^ T lymphocytes, unactivated natural killer cells, activated natural killer cells, unactivated mast cells, activated mast cells, resting dendritic cells, activated dendritic cells, neutrophils, eosinophils. Box plots showing trends in the abundance of different immune cell infiltrates between the two groups, based on a *p* < .05 screening sample.

### Assessment of DEGs and functional enrichment in two groups of SKCM

On the two SKCM datasets, differential gene expression analysis was performed using the statistical software R4.1.3. Differentially expressed genes (DEGs) were identified using a *p*-value adj. *p-*value .05 as a screening condition and visualized as a heat map in R. The DAVID (Database for Annotation, Visualisation, and Integrated Discovery) database (https://david.ncifc.rf.gov/) was used to perform GO functional annotation and KEGG pathway analysis on the aforementioned common DEGs. The DAVID database combines biological data and analysis techniques to provide researchers with gene and protein annotation capabilities. GO is a bioinformatics tool that is used to analyze and annotate biological processes in genes. GO is divided into three parts: molecular function, biological processes, and cellular components. KEGG analysis allows one to analyze signaling pathways from large scale molecular datasets generated by high throughput experimental techniques, including multiple protein interactions and processes that together regulate cellular function and metabolic activity. For threshold screening, an adjusted *p* .05 is used, and the results are visualized using the R language ggplot2 package.

### Multi-factor cox prognostic regression model

A multi-factor cox regression analysis was used to screen variables for significant prognosis, and a multi-factor cox regression analysis was built to model the risk of immunogenesis, with each patient receiving a risk score based on the discovered formula.

### Statistical analysis

R was used for all statistical analyses. Cox regression analyses were carried out using the R packages survival and survminer for one-way and multi-way cox regression, with *p*-value .05 set as the prognostic significant variable.

## Results

### Identification of DEGs in melanoma patients

**FIGURE 1:**
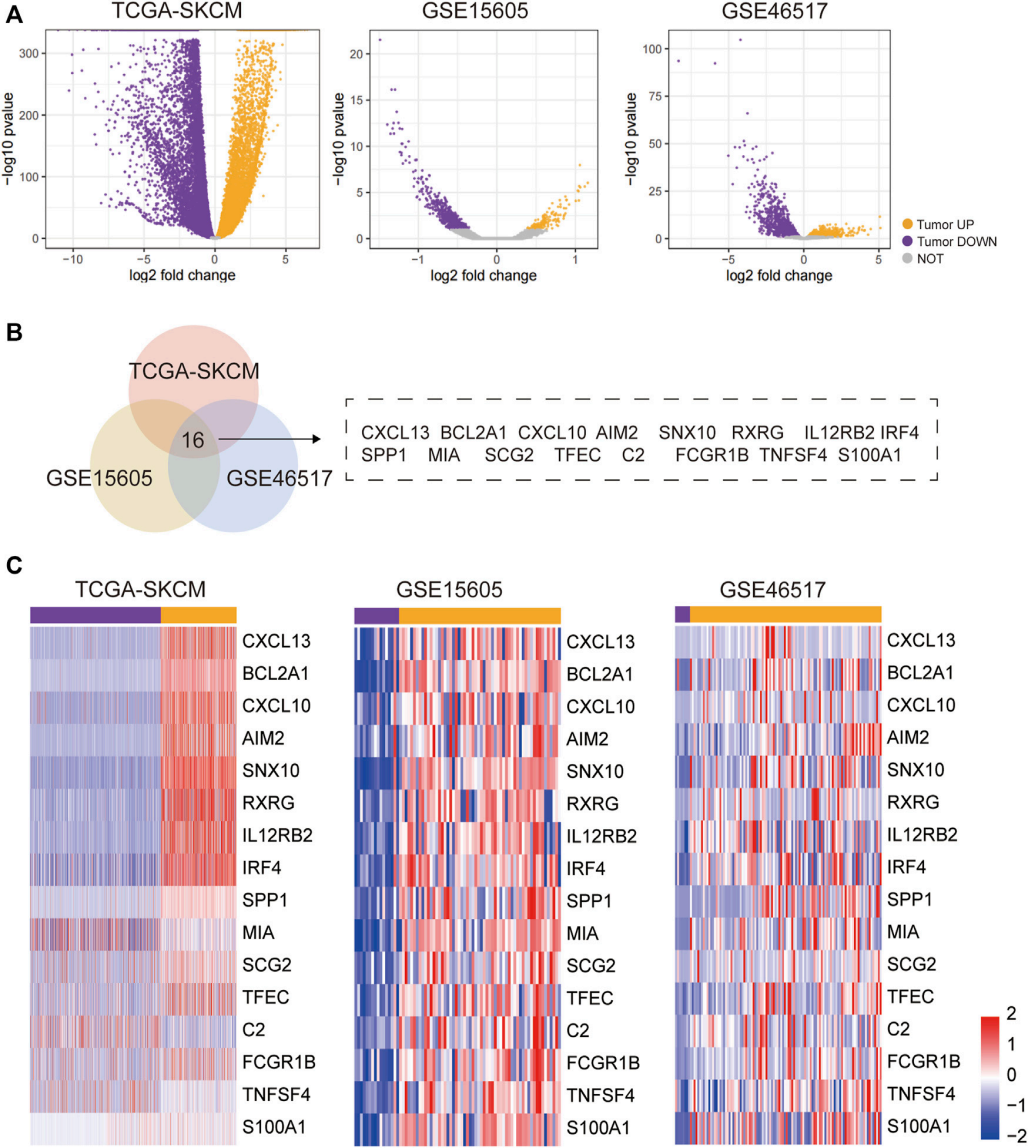
DEG identification and analysis. **(A)**Volcano plot of DEGs in TCGA-SKCM, GSE15605, and GSE46517 datasets. **(B)** Venn diagram: Venn plots of DEGs in the TCGA-SKCM, GSE15605, and GSE46517 datasets, with the overlapped part representing the 16 common DEGs in the three data sets. **(C)** The 16 overlapping parts portray the DEGs that are shared by the three data sets.

After screening the inclusion conditions, DEGs were identified in SKCM and normal control skin using the GSE15605, GSE46517, and TCGA datasets. The same methods and thresholds were used to analyse the differences between tumour and normal samples from GSE15605 and tumour and normal samples from GSE46517, and to plot the volcanoes. The tumour samples from GSE15605 had 156 upregulated genes; 564 downregulated genes; and the tumour samples from GSE46517 had 1,634 upregulated genes; 1894 down-regulated genes. All genes in the three datasets are plotted as volcanoes (Figure 1A), with blue representing upregulation and red representing downregulation. To better understand the distribution of DEGs, a Venn diagram was plotted separately from the visualised heat map using the online software Draw Venn Diagram (Figures 1B,C).

### Differentiation of two subtypes of cutaneous melanoma

**FIGURE 2:**
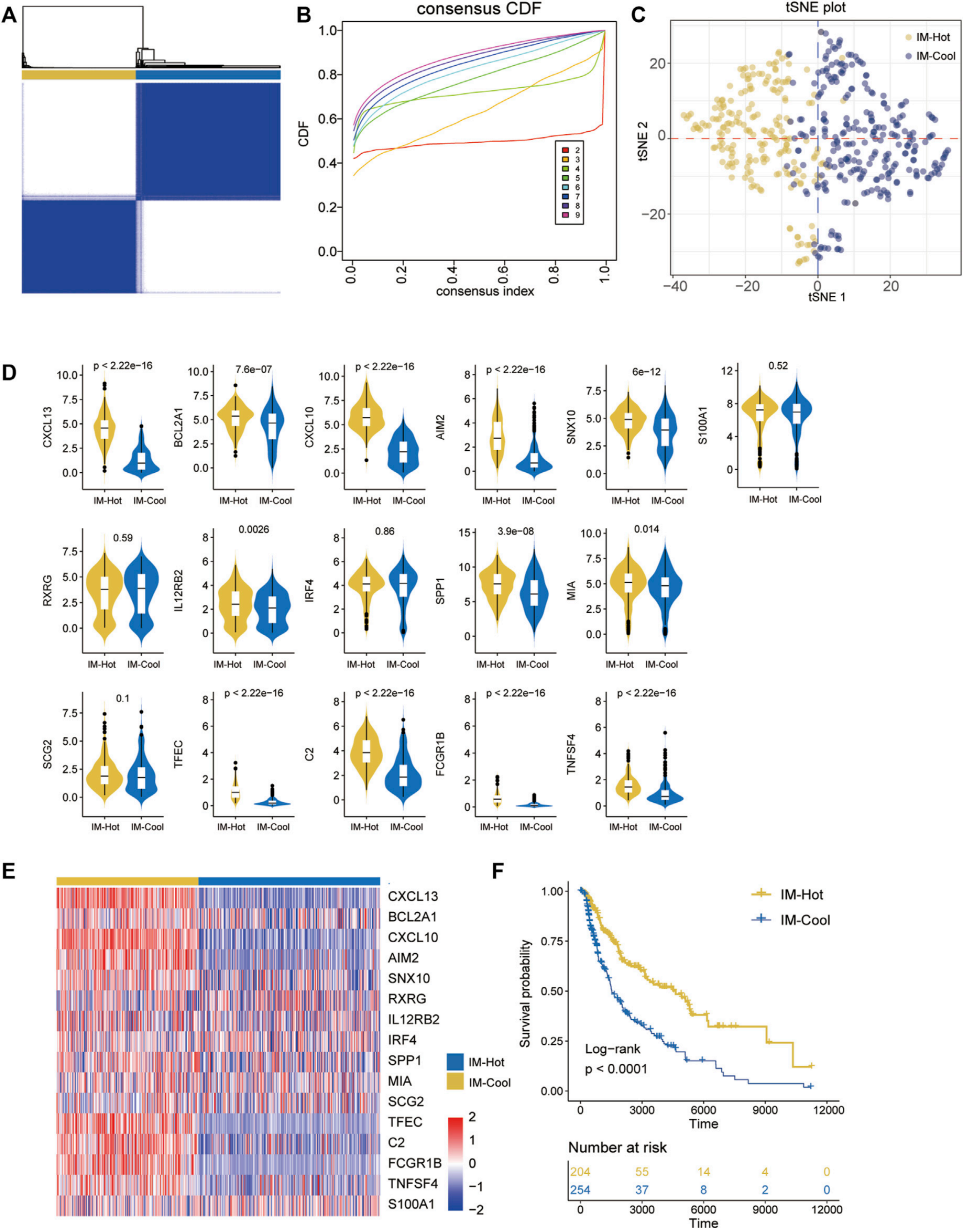
Identification of immune-related SKCM subtypes in the TCGA cohort. **(A)** The heatmap depicts the consensus matrix at k = 2 in the TCGA group. **(B)**The cumulative distribution function (CDF) curves in consensus cluster analysis. Consensus scores for different subtype numbers (k = 2–9) are presented. **(C)**The stratification into three subtypes validated by t-SNE in TCGA cohorts. Each dot represents a single sample, and each color denotes a subtype. **(D)** Box figure:the 16 overlapped part represents the common DEGs in the two TCGA cohorts.**(E)** heatmap:the 16 overlapped part represents the common DEGs in the two TCGA cohorts.**(F)** Survival analysis of patients with the three diffuse glioma subtypes (IM-Hot and IM-Cool) in TCGA cohorts. The log-rank test was conducted to determine the significance of the differences.

Based on 16 overlap genes, 472 SKCM patients were classified into different subtypes (K = 2, 3, 4, 5, 6, 7, 8, and 9) using unsupervised consensus clustering analysis. Molecular typing was performed on the TCGA-SKCM samples to obtain the results shown in Figure 1. The best division (k = 2) was selected as the optimal number of clusters based on the CDF curve (Figures 2A,B), and two Immune-related Clusters were identified, including the IM-Hot Cluster and the IM-Cool Cluster. t-SNE was performed to reduce the dimensionality in order to verify the assignment of subtypes. The two-dimensional t-SNE distribution pattern was confirmed to be robustly consistent with the CDF curve (Figure 2C), a result that suggests that the two sets of samples have been successfully separated. Among the two Immune-related Clusters, the IM-Hot Cluster upregulated most of the immune-related genes and showed significant immunoreactivity, while the IM-Cool Cluster showed relatively low immunoreactivity (Figures 2D,E). We also explored the differences in survival information between the two groups; the survival curves showed a significant difference in overall survival between the two groups (*p* < .0001); with the IM-Cool group having a significantly worse prognosis; suggesting that the immune response in the IM-Hot group favoured the onset of anti-tumour activity and thus the patient’s prognosis, while the IM-Cool group was not as active due to the immune response or was suppressed by tumour cells. The IM-Cool group had a significantly worse prognosis because the immune response was not active or was suppressed by the tumour cells (Figure 2F).

### Acquisition of immune pathway gene sets related to immune pathway gene

**FIGURE 3:**
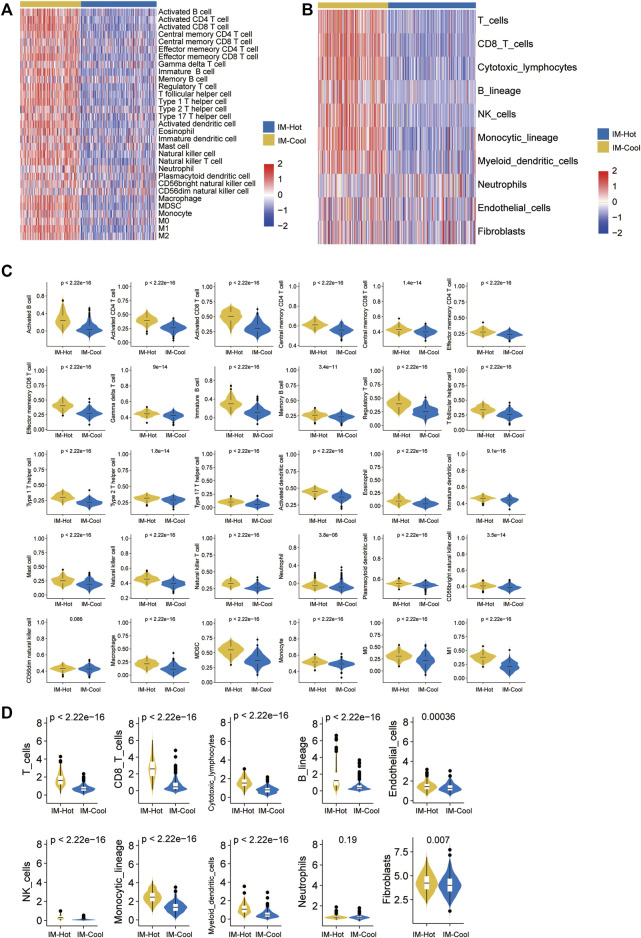
Immune characteristics of the two subtypes in TCGA cohort. **(A,B)** The heatmap showing the abundance of immune-cell populations calculated by GSVA and Mcpcounter in the two subtypes. **(C,D)** Box plots show trends in the abundance of different immune cell infiltrations between the two subtypes in TCGA cohort.

Given the significance of TME in tumour growth and treatment, we investigated it using a variety of immune assessment algorithms. First, GSVA enrichment analysis revealed that SKCM patients in the IM-Hot group have been strongly associated with the Activated B cell pathway, Activated CD4 T cells, Activated CD8 T cells, Eosinophil, Immature dendritic cell, Mast cell natural killer cell, Plasmacytoid dendritic cell, CD56bright natural killer cell, Macrophage, and other immune pathways and functions (Figure 3A). We then used the Mcpcounter (R package IOBR) algorithm to evaluate the differences in immune cell intrusion between the two groups, and the heatmap depicts the results. Compared to the IM-Cool Cluster, the IM-Hot Cluster had higher expression levels of T_cells, Cytotoxic_lymphocytes, B_lineage, NK_cells, Monocytic_lineage, Myeloid_dendritic_cells, and Neutrophils expression levels were significantly higher in the IM-Hot group than in the IM-Cool group (Figure 3B), consistent with the findings of the GSVA analysis The IM-Hot group’s strong immune response was affirmed. The CIBERSORT algorithm was then used to analyze the classes and ratios of the 30 immune cells. Violins were used to represent the relative proportions of the 30 immune cells in the IM-Cool Cluster and IM-Hot Cluster, and the results revealed significant differences in immune cell distribution according to the risk model. Furthermore, when compared to IM-Hot Cluster, we concluded that, with the exception of CD56dim natural killer cells, which did not vary statistical significant between both the two groups (*p* > .05), the remaining immune cells such as T-cells, B-lineage, cytotoxic-lymphocytes, and NK-cells showed high expression in the IM-Hot Cluster (*p* < .05) (Figures 3C,D). The results showed that immune cell infiltration was significantly richer in the IM-Hot group than in the IM-Cool group.

### Differential analysis and functional enrichment analysis between the IM-Hot and IM-Cool groups

**FIGURE 4:**
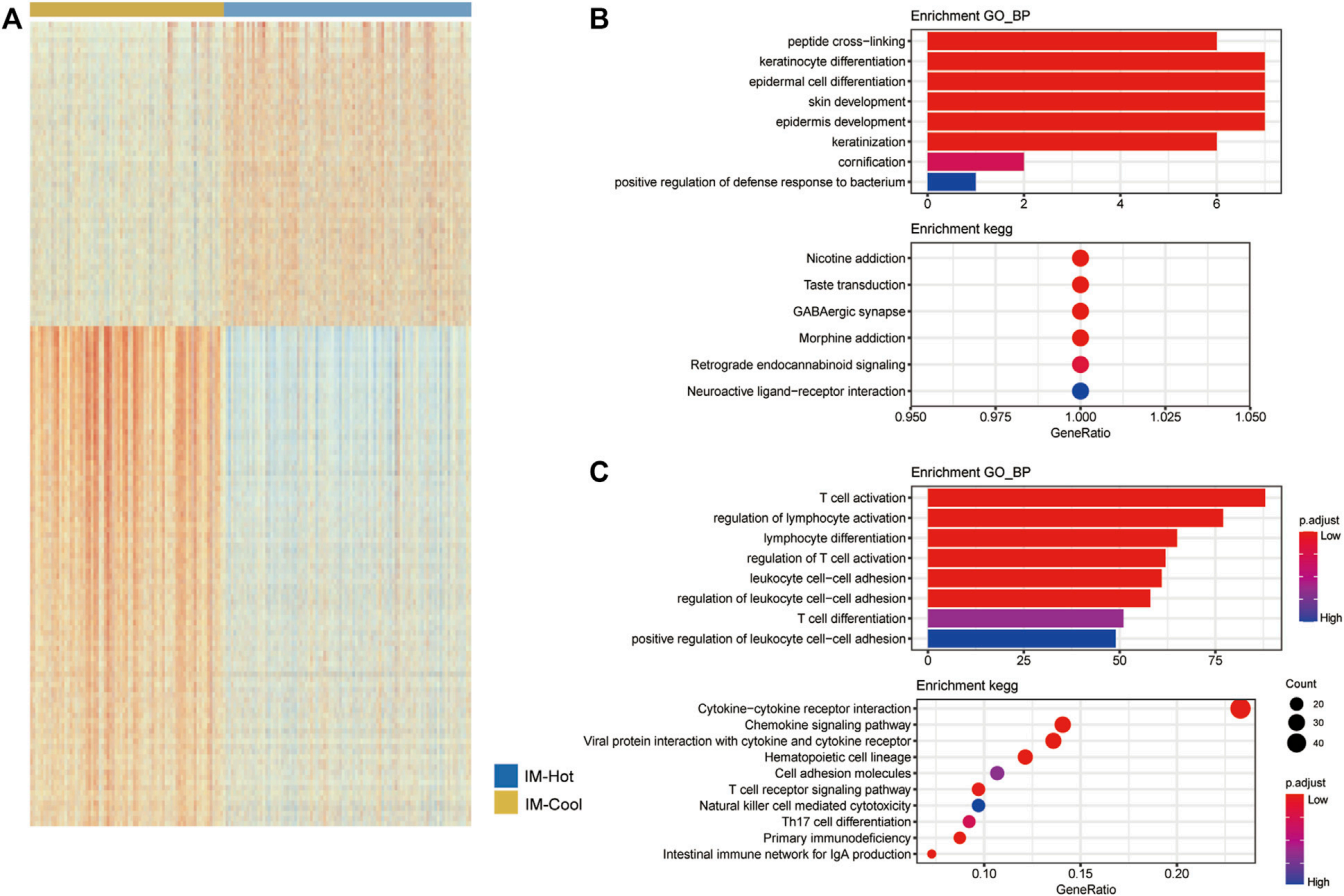
GO and KEGG analysis results of genes. **(A)** The heatmap showing the DEGs in the two subtype TCGA cohorts. **(B)** Up-regulated genes in the IM-Hot group GO function enrich-ment results, and KEGG pathway enrichment results. **(C)** Up-regulated genes in the IM-Cool group GO function enrichment results, and KEGG pathway enrichment results.

We performed differential analysis of the two melanoma subtypes to identify specific differentially expressed genes and signalling pathways. The genes were significantly differentially expressed when absolute |logFC| > 0 and FDR <0.1. (Figure 4A). We further performed GO functional enrichment analysis and KEGG pathway enrichment analysis for the up-regulated genes in the IM-Hot group and the IM-Cool group, respectively. As shown in the figure, the up-regulated DEG in the IM-Hot group compared to IM-Cool, GO BP was mainly enriched in peptide cross-linking, keratinocyte differentiation, epidermal cell differentiation, skin development, epidermis development, keratinization, cornification. KEGG: Nicotine addiction, Taste transduction, GABAergic synapse, Morphine addiction, Retrograde endocannabinoid signaling, Neuroactive ligand-receptor interaction (Figure 4B). IM-Hot group compared to the down-regulated DEG in the IM-Cool, GO BP was mainly enriched in T cell activation, regulation of lymphocyte activation, lymphocyte differentiation, regulation of T cell activation, leukocyte cell-cell adhesion, regulation of leukocyte cell-cell adhesion, T cell differentiation, positive regulation of leukocyte cell-cell adhesion. KEGG: Cytokine−cytokine receptor interaction, Chemokine signaling pathway, Viral protein interaction with cytokine and cytokine receptor, Hematopoietic cell lineage, Cell adhesion molecules, T cell receptor signaling pathway, Natural killer cell mediated cytotoxicity, Th17 cell differentiation, Primary immunodeficiency, Intestinal immune network for IgA production (Figure 4C).

### Construction of a risk model for copper immunoreactivity-related genes in cutaneous melanoma

Although the results of this study can predict the prognosis of SKCM patients, these studies are based on patient populations and therefore cannot accurately predict the immune activation status of individual patients. Therefore, we constructed a prognostic model for SKCM by basing it on the expression levels of immune activation-related genes.

**FIGURE 5:**
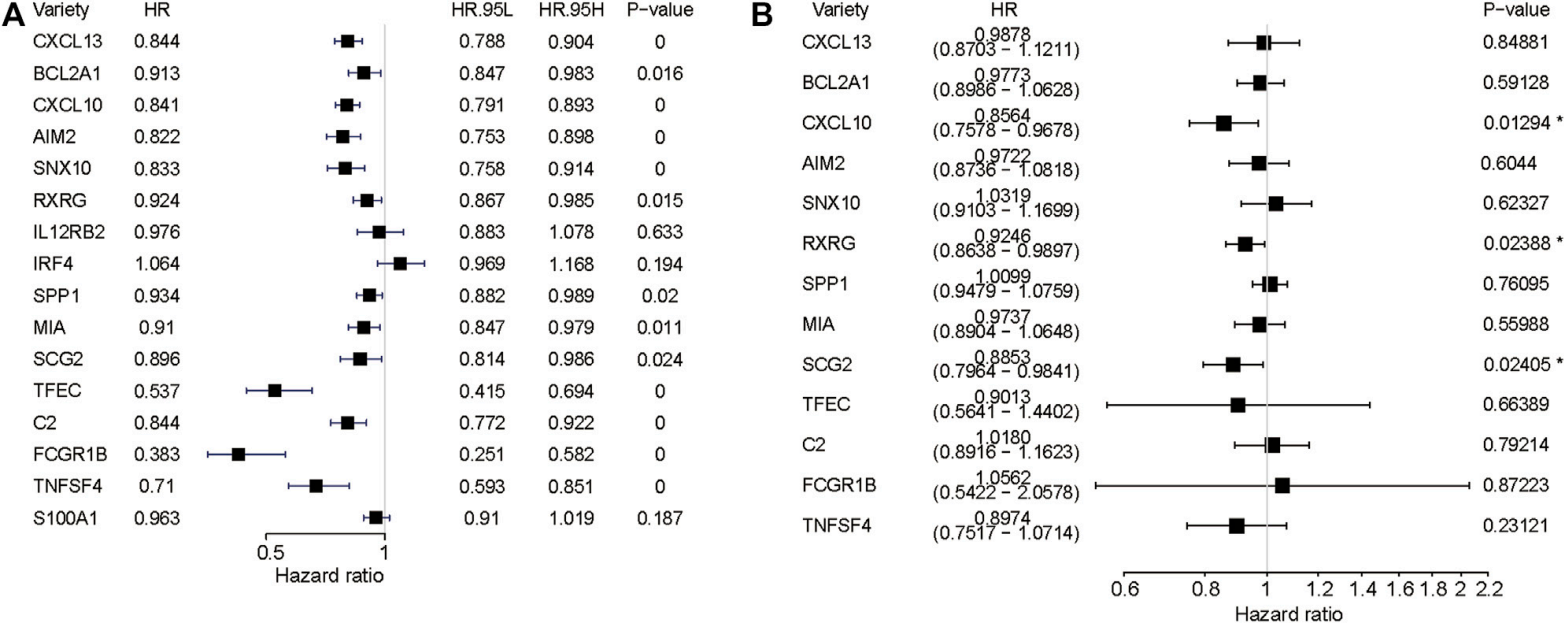
Regression analysis. **(A)** Forest plots depicting the univariate Cox regression analysis. **(B)**multivariate Cox regression analysis.

Univariate Cox regression combined with multifactorial cox regression was used to analyse 16 genes (Figures 5A,B), and three genes (CXCL10, RXRG, SCG2) were finally screened. Based on the expression of these three genes and their corresponding regression coefficients, Risk score = 0.924583*exp (RXRG) + 0.885278*exp (SCG2) − 0.155031*exp (CXCL10).

**FIGURE 6:**
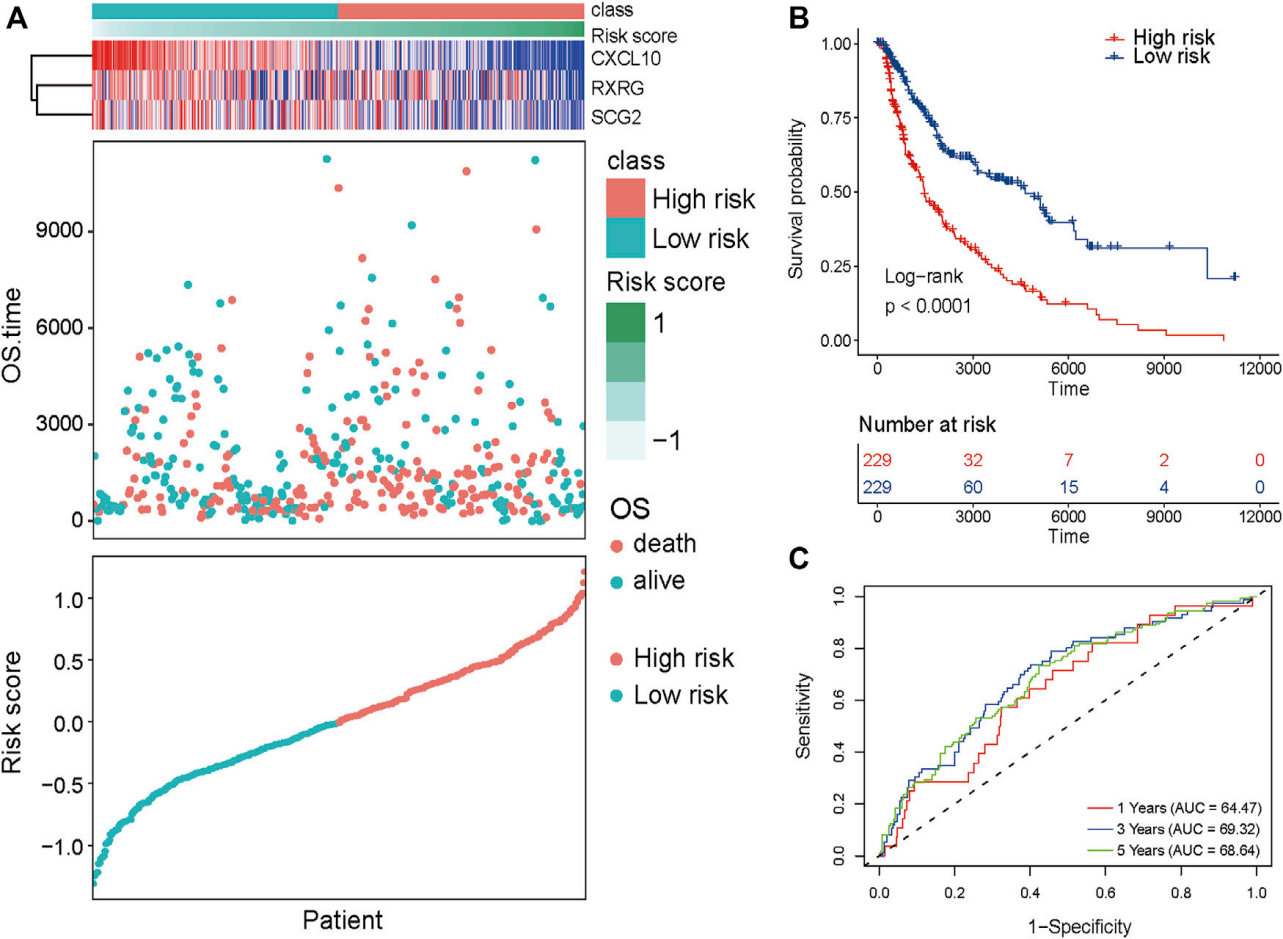
Prognostic value of the proposed subtyping for SK CM. **(A)** Risk Score, and expression of 3-gene in TCGA training set. **(B)** KM survival curve distribution of 8-gene signature in training set. **(C)** ROC curve of 3-gene signature classification and AUC.

SKCM patients were divided into high-risk and low-risk groups based on the critical value of the median risk score (Figure 6A). k-M survival analysis showed that the prognosis of SKCM patients was significantly correlated with the risk score, and the survival curves demonstrated a significant difference in overall survival between the two groups (*p* < .0001, R package survivor (Figure 6B); survminer); with the high-risk group having a significantly worse prognosis. The ROC curve was used to assess the accuracy of the prediction model. The area under the ROC curve at 1, 3, and 5 years was 0.6447, 0.6932, and 6,864 respectively, indicating that the risk score can be used to reliably predict the prognosis of patients with SKCM (Figure 6C).

## Discussion

Despite significant progress in SKCM security check, diagnosis, and therapeutic interventions, the prognosis of advanced malignant SKCM remains poor (Zhao et al., 2019). Given that the lack of quick and efficient diagnostic tools and early metastases characteristics is the primary cause of this poor prognosis and mortality rates, we decided to use bioinformatics analysis to find effective biomarkers for early diagnosis.

Changes inside the cellular immune microenvironment (TME) have been discovered to play a critical role in regulating the progression of various cancers and can affect the prognosis of SKCM in recent years. In yang’s study, lower immune cell activity was linked to a worse prognosis in SKCM (Yang et al., 2022). Furthermore, Su’s research demonstrated that increased immune activity can successfully inhibit tumor progression (Su et al., 2022) and is an extremely promising cancer strategy for treating. Meanwhile, a growing number of studies have revealed that the immune cell transcriptome is important in the biology of SKCM as a starting to emerge molecular and genetic biomarker (Wang et al., 2020). There have been no studies on the link between immune signalling pathway genes and the prognosis of SKCM. We decided to investigate how immune activation-related genetic traits regulate the immunologic process and thus influence tumour growth and patient prognosis in SKCM based on the scientific backstory described above.

Bioinformatic analyses were performed on normal skin samples and SKCM samples based on the datasets GSE15605, GSE46517, TCGA, and GTEx databases. In this study, differential analysis of tumour samples from TCGA-SKCM and normal samples from GTEX SKIN; yielded 10,917 upregulated genes in tumour samples; 10,734 downregulated genes; 156 up-regulated genes in tumour samples from GSE15605; 564 downregulated genes; 1,634 upregulated genes in tumour samples from GSE46517.1894 downregulated genes; doing intersection of upregulated genes in tumor samples from the three data sets, 16 overlap genes were obtained, including CXCL13, BCL2A1, CXCL10, AIM2, SNX10, RXRG, IL12RB2, IRF4, SPP1, MIA SCG2, TFEC, C2, FCGR1B, TNFSF4, and S100A1, all of these 16 genes were upregulated in tumour samples in different datasets, indicating that these 16 genes are widely activated in tumours and significantly enriched for biological processes related to the immune response, suggesting activation of the immune response in SKCM tumours.

Consensus clustering (R package ConsensusClusterPlus) was done on TCGA-SKCM samples using 16 overlap genes to molecularly typify SKCM for tumours; all samples were divided into a total of two phenotypes. tSNE plots show that the two class curves are the flattest curves, indicating that class 2 is the optimal classification. tSNE plots show that the two phenotypes we divided phenotypes are clearly distinguishable in the low-dimensional space, indicating the robustness of the typing (R package Rtsne). The box plots show a consistent expression trend for most of the 16 genes in the two typologies, i.e. significantly higher in one group than in the other; we therefore named the two typologies Immune-Hot and Immune-Cool; IM-Hot significantly upregulates most of the immune-related genes, indicating activation of immune activity, while IM-Cool Cool; we explored differences in survival information between the two groups; survival curves showed significant differences in overall survival between the two groups (*p* < .0001, R package survivor; survminer); with the IM-Cool group having a significantly worse prognosis; suggesting that the immune response in the IM-Hot group favours anti-tumour activity, which in turn favoured patient prognosis; whereas the IM-Cool group had a worse patient prognosis due to an inactive immune response, or suppression by tumour cells.

Immune cell infiltration in both groups was assessed by both single sample enrichment analysis (R package GSVA) as well as Mcpcounter (R package IOBR); the heat map shows the trend of different immune cell infiltration abundance between the two groups; the results indicate that immune cell activity and infiltration abundance were significantly higher in the IM-Hot group than in the IM-Cool group; further confirming the high immune response in the IM-Hot group; further confirming the high immune response in the IM-Hot group. Box plots show the trend of different immune cell infiltration enrichment between the two groups; the results are consistent with the immune heat map for differential analysis, GO functional enrichment analysis and KEGG pathway enrichment analysis (R package clusterProfiler, adjust *p*-value < .05) between the IM-Hot and IM-Cool groups. Upregulated genes in the IM-Hot group were enriched in T cell immunity and immunochemokine-related pathways and functions, indicating extensive activation of T cell antitumour immunity in the IM-Hot group.

Finally, using univariate Cox, LASSO, and multivariate Cox regression analyses, three protective innate immune genes were identified to build a risk predictive model. This stepwise downscaling approach to ultimately screen out prognostic factors critical genes and then use them to build a risk model has been noted in many great articles and is reliable (Jiang et al., 2022). Subsequent studies have confirmed the risk model’s outstanding predictive power. In addition, we developed a precise trend line to better predict SKCM patients’ 1-, 3-, and 5-year survival rates. We were shocked to learn that all three immunologic activation genes (CXCL10, RXRG, and SCG2) have been linked to cancer progression. According to previous research, nanoparticle shipment of CXCL10 plasmids helps promote T cell incursion in tumors and synergizes with anti-PD1 immunoglobulin activity (Ma et al., 2022). Kawaguchi et al. (2022) discovered a significant rise in CXC motif chemokine ligand 10 (CXCL10) mRNA levels as well as CTL infiltration in tumors. Liu et al. (2022), showed that upregulation of Li et al. (2017) showed that CXCL10/CXCL11 levels significantly enhanced CD8^+^ TIL recruitment in tumour tissue. High concentrations of CXCL10 were deposited in HCM identified by antibody arrays, which may help to predict clinical outcome in HCC patients. In addition, Schulten et al. (2015) showed that RXRG was significantly upregulated in follicular FVPTCs of papillary thyroid carcinoma. Smetannikova et al. (2019) reported that results of differential expression in DNA samples from lung cancer *versus* normal lung tissue showed that RXRG had high sensitivity and specificity and possessed good diagnostic potential. This is consistent with our findings, indicating that RXRG is essential in the advancement of SKCM. Notably, SCG2 has been implicated in the progression of colon cancer and straightforward cell carcinoma of the kidneys. Wang et al. (2022) proved that SCG2 is a significant predictor forecaster of colorectal cancer using several functional assays (CRC). In CRC patients, high SCG2 utterance was associated with poor survival and advanced clinical stage. SCG2 may start regulating numerous cancer and immune-related pathways in CRC, influencing tumor immunity by trying to regulate immune cell infiltration and monocyte polarization. SCG2 was identified as a particular antigen for straightforward cell renal cell carcinoma (ccRCC) and not only was associated with a poor prognosis but was also strongly associated with immune cells and immune roadblocks, according to Xu’s research (Xu et al., 2022b). Roudi et al. evaluated the genomic expression profile of D10 melanoma cells. In CD133 (+) D10 cells, many genes related to tumor aggressiveness were upregulated, including some of the genes in this study (Roudi et al., 2015). Our results suggest that CXCL10 may play a protective factor in lowering the risk of SKCM and trying to improve prognosis, while RXRG, SCG2 was affiliated with a poor prognosis in SKCM, a trying to find in line with previous reports of similar research, with resulting methods to be investigated further. We discovered that the lymphocytes and immunity in the IM-Hot group were greater than the intrusion status in the IM-Hot group, and the diagnosis was better. It is recommended that the greater standard of immune penetration may be related to the better prognosis of patients in the low-risk group. These findings imply that the reporting tool we developed is heavily influenced by the immune landscape of the SKCM micro—environment. This also implies that immune cell descriptor and organ function activation could indeed influence tumor prognosis. More research is required to discover the molecular mechanisms underlying SKCM immunity.

This research investigated the prognostic role of immunological pathways in the prognosis of SKCM tumour cells. We built a model for predicting the prognosis of SKCM based on immune pathway genes and TCGA data from SKCM patients, which gives promise for SKCM therapy and diagnosis.

## Data Availability

The original contributions presented in the study are included in the article/Supplementary Material, further inquiries can be directed to the corresponding author.
